# Fundamentals in Covid-19-Associated Thrombosis: Molecular and Cellular Aspects

**DOI:** 10.3389/fcvm.2021.785738

**Published:** 2021-12-17

**Authors:** Daniella M. Mizurini, Eugenio D. Hottz, Patrícia T. Bozza, Robson Q. Monteiro

**Affiliations:** ^1^Institute of Medical Biochemistry Leopoldo de Meis, Federal University of Rio de Janeiro (UFRJ), Rio de Janeiro, Brazil; ^2^Oswaldo Cruz Foundation, Laboratory of Immunopharmacology, Oswaldo Cruz Institute, Rio de Janeiro, Brazil; ^3^Laboratory of Immunothrombosis, Department of Biochemistry, Federal University of Juiz de Fora (UFJF), Juiz de Fora, Brazil

**Keywords:** COVID-19, thrombosis, platelets, monocytes, neutrophil extracellular trap, endothelium, blood coagulation

## Abstract

The novel coronavirus disease (COVID-19) is associated with a high incidence of coagulopathy and venous thromboembolism that may contribute to the worsening of the clinical outcome in affected patients. Marked increased D-dimer levels are the most common laboratory finding and have been repeatedly reported in critically ill COVID-19 patients. The infection caused by Severe Acute Respiratory Syndrome Coronavirus-2 (SARS-CoV-2) is followed by a massive release of pro-inflammatory cytokines, which mediate the activation of endothelial cells, platelets, monocytes, and neutrophils in the vasculature. In this context, COVID-19-associated thrombosis is a complex process that seems to engage vascular cells along with soluble plasma factors, including the coagulation cascade, and complement system that contribute to the establishment of the prothrombotic state. In this review, we summarize the main findings concerning the cellular mechanisms proposed for the establishment of COVID-19-associated thrombosis.

## General Features of Hemostatic Imbalance in Covid-19 Progression

Since the first reports comparing mild and severe covid COVID-19 patients, it was clear that the more severe illness was strongly associated with hypercoagulability (1, 2). COVID-19-associated coagulopathy has been consistently reported in patients admitted to hospital, in particular those severely ill. The most frequently reported laboratory findings related to COVID-19 coagulopathy include increased D-dimer levels and fibrinogen concentration, decreased platelet count, and slightly prolonged clotting time (3–5). A meta-analysis identified significantly lower platelet count in COVID-19 patients, and showed an association between thrombocytopenia, disease severity and increased risk of mortality (3). Fibrinogen levels are commonly high in the early stage of COVID-19 infection; however, a decline in plasma fibrinogen levels was observed in the late stage of the disease among non-survivors (5, 6). On the subject of coagulation tests, it has been demonstrated that COVID-19 patients have a modest prolongation of prothrombin time (PT) and activated partial thromboplastin time (APTT), although a marked increase in the D-dimer levels has been repeatedly reported (5, 7). An increasing number of studies have shown that D-dimer is commonly elevated in COVID-19 patients and that increased levels of this coagulation marker are associated with poor outcomes (2, 5, 8). In this context, Tachil et al. early proposed D-dimer concentrations as essential markers of COVID-19 severity (8). Other authors have proposed progressive stages of COVID-19–associated hemostatic abnormalities that mostly rely on D-dimer levels and could define the antithrombotic therapy as well as additional patient management procedures (9, 10). As the pandemic continues, it has become clear that COVID-19 progresses with increased thrombosis propensity being a significant cause of death in hospitalized patients (11–14). In this context, both arterial and venous thrombosis have been reported (15, 16). In addition, post-mortem analyses have shown microvascular thrombi in the lung, kidney, and heart of COVID-19 patients (17). Interestingly, histology of lung and skin samples revealed generalized thrombotic microvascular injury accompanied by the presence of complement deposits, more specifically C5b-9 complement complex and C4d complement split factor (18). The complement system is an important part of the innate defense against common pathogens, and its activation has previously been described in respiratory viral infections like influenza A and SARS-CoV (19). Additional evidence of complement activation following SARS-CoV-2 infection includes the observation of increased plasma levels of complement markers, such as sC5b-9, C5a, and C3a, in COVID-19 patients compared to non-COVID-19 and healthy controls, and their association with disease severity (20–22). Considering that a crosstalk between complement activation and coagulation system has long been documented, the recent findings open debate on whether complement activation triggered by COVID-19 contributes to COVID-19-associated thrombosis. Whether the prothrombotic phenotype observed in COVID-19 patients is a combination of hypercoagulability and impaired fibrinolysis has been one of the theories of several clinical studies. Recent systematic reviews have found a decreased fibrinolytic capacity in COVID-19 patients (23, 24). Some studies reported a reduced fibrinolytic activity in COVID-19 patients in intensive care unit (ICU) compared with non-ICU COVID-19 patients, and in patients with thrombotic events among those requiring ICU care (23). Using diverse viscoelastic tests, different research groups were able to detect impaired fibrinolysis in blood samples from COVID-19 patients, despite a contradictory increase in circulating levels of D-dimer (23, 24).

In this review, we summarize the main findings concerning the mechanisms proposed for the establishment of COVID-19-associated thrombosis, focusing on the involvement of cellular components of the clotting and immune system.

## Endothelium

The vascular endothelium is a highly specialized and dynamic organ that exerts a number of important functions that are critical to maintaining adequate blood supply to vital organs (25). Some of these functions include control of hemostasis as well as regulation of vascular tonus and permeability (25–28). In addition to regulating the systemic blood flow, the endothelium actively participates in both the innate and adaptive immune responses (29).

Viral pathogens, including respiratory viruses, members of the herpesvirus family, and human immunodeficiency virus (HIV) can damage the endothelium, leading to detrimental shifts in the vascular equilibrium toward inflammation, vasoconstriction and procoagulant activity (30–34). Correspondingly, the autopsy of lung samples from different cohorts of COVID-19 patients revealed the presence of viral inclusions in endothelial cells (35, 36). In addition to the evidence of direct viral infection of endothelial cells, post-mortem histology also disclosed the presence of endothelial inflammation (35, 36). Immunohistochemical analysis of lung specimens revealed staining patterns consistent with apoptotic endothelial cells and mononuclear cell infiltrates (36). Indeed, SARS-CoV-2 infected endothelial cells were also detected in several organs besides the lung, including the kidney, heart, and liver (36–38).

There is ample evidence that SARS-CoV-2 uses the functional receptor for the SARS-CoV virus, the angiotensin-converting enzyme 2 (ACE2) receptor, to infect the host cells (39–41). ACE2 is a type I integral transmembrane protein widely expressed in human tissues in addition to the lungs (42). Interestingly, ACE2 has been detected in arterial and venous endothelial cells in a variety of human tissues, including the nasal mucosa, lung, small intestine, kidney, and brain (42). Similar to what has been reported to other coronaviruses, the cell entry of SARS-CoV-2 also depends on cell surface proteases which cleave the Spike protein, allowing the fusion of the viral and cellular membranes (39, 41, 43). Recent studies suggest that both SARS-CoV and SARS-CoV-2 employ the protease transmembrane protease serine 2 (TMPRSS-2), which is also expressed in human endothelial cells (39, 42). These findings support the hypothesis that SARS-CoV-2 can directly infect blood vessel cells, which was recently confirmed by Monteil et al. (40).

Besides the direct effects of SARS-CoV-2 on endothelial integrity, evidence from recent studies points that endothelial cells can be activated by humoral factors presented in the blood of SARS-CoV-2 infected patients (44, 45). Stimulation of human umbilical vein endothelial cells (HUVECs) with plasma or sera from COVID-19 patients resulted in activation of the endothelial cells, determined by overexpression of adhesion molecules and upregulation of thromboinflammatory genes associated with downregulation of antithrombotic genes (44, 45). This shift toward a prothrombotic surface appears to be triggered by alterations in the Tie2-angiopoietin axis (44). Tie2, or angiopoietin receptor 2, is a tyrosine kinase receptor selectively expressed on vascular endothelial cells, and its activation by angiopoietin 1 maintains the endothelial quiescence and suppresses vessel inflammation (46). It has been experimentally demonstrated that pharmacological activation of Tie2 effectively reversed the expression of thromboinflammatory genes induced by COVID-19 plasma *in vitro* (44). Another mechanism whereby COVID-19 may induce activation of endothelial cells appears to be mediated by antiphospholipid (aPL) antibodies. aPL antibodies comprise a heterogeneous population of autoantibodies directed against phospholipids and phospholipid-binding proteins and are associated with a higher risk of thrombosis in COVID-19 (47). A high prevalence of antiphospholipid antibodies has been reported in COVID-19 patients (12, 47), and the presence of different aPL correlates with endothelial activation (45). Positive aPL sera from COVID-19 patients induced upregulation of the cell adhesion molecules E-selectin, vascular cell adhesion molecule 1 (VCAM-1), and intracellular adhesion molecule 1 (ICAM-1) in HUVECs, which was abolished by total IgG depletion from the COVID-19 sera (45).

It is well-documented that activated endothelial cells evolve from an antithrombotic phenotype to a prothrombotic state (48). Under normal circumstances, endothelial cells possess anti-coagulant functions by expressing several molecules that limit or inhibit the coagulation process, however after inflammatory stimuli (e.g., interleukin (IL)-1 and tumor necrosis factor (TNF)-α), these cells acquire pro-coagulant functions, such as shedding of microparticles with exposed phosphatidylserine (PS) that enable the assembly of clotting enzyme complexes and the generation of thrombin (49, 50). Interestingly, activation of HUVECs with plasma from severe COVID-19 patients incited externalization of PS, creating a suitable surface for the assembly of coagulation complexes on the endothelium (44). Indeed, generation of factor Xa and thrombin were detected in HUVECs following exposure to COVID-19 plasma (44). Nevertheless, IL-1 and TNF-α, cytokines that are markedly increased in COVID-19 patients, also induce endothelial cells to synthesize tissue factor (TF), the primary initiator of coagulation (51, 52). TF is a transmembrane cellular receptor for the plasma coagulation factor VIIa (FVIIa), and the binding of FVIIa to TF is the initial step of a sequential proteolytic cleavage of coagulation factors that results in thrombin generation, platelet activation, and clot formation (53).

Endothelial cell dysfunction commonly observed in COVID-19 patients may be a result of pro-inflammatory cytokines that are produced in response to viral infection (54, 55). There is strong evidence that severe SARS-CoV-2 infection is accompanied by a massive inflammatory response resulting in the release of a large number of pro-inflammatory cytokines (56–59). The resultant increase in the pro-inflammatory cytokine levels might result in the loss of the antithrombotic phenotype of the endothelium, occasioning the activation of the coagulation cascade, platelets, and complement system in the vasculature (26, 48). For example, it is well-known that IL-6 secreted by immune and endothelial cells in response to a viral infection plays an important role in activating the endothelium during the early phase of inflammation (60). This cytokine not merely increases the vascular permeability but also promotes the secretion of pro-inflammatory cytokines by endothelial cells, sustaining an amplification loop that contributes to the excessive cytokine production, one of the most prominent characteristics of COVID-19 (61, 62).

Endothelial cell activation triggered by pro-inflammatory cytokines is an initial step in the platelet plug formation. It is well-known that endothelial cells store von Willebrand factor (VWF) and surface adhesion molecules, including P-selectin in intracellular granules, called Weibel–Palade bodies, which are then released upon endothelial cell activation (48, 63–65). Elevated levels of both VWF antigen and VWF activity were detected in the plasma of COVID-19 patients (with the higher values being observed among patients requiring intensive care), corroborating the previous observations of endothelial cell activation elicited by SARS-CoV-2 infection (66, 67). However, VWF activity depends on its size, that is, the larger the length of the VWF multimer, the greater its ability to adhere to platelets and other blood cells (68). Under normal circumstances, ultra-large VWF multimeters are cleaved by ADAMTS13 (a disintegrin and metalloproteinase with thrombospondin type 1 motifs, member 13), preventing thrombus formation (69). The deficiency of ADAMTS13, which was also observed in blood samples from COVID-19 patients, results in disseminated microvascular thrombosis, characteristic of thrombotic thrombocytopenic purpura (TTP) (67, 70). There is evidence that, in COVID-19 patients, the increase in VWF levels coexist with a moderate reduction in ADAMTS13 activity (67).

## Platelets

Platelets are anucleate blood cells generated in the bone marrow from precursor cells called megakaryocytes and are well-known for their essential role in hemostasis and thrombosis [reviewed in (71)]. Low platelet count and altered morphology are often observed during viral infections (72, 73). The low number of circulating platelets following a viral infection might be a result of either decreased platelet production or increased platelet destruction (72). Usually, the reduction in platelet production is observed at later stages of infection, whereas an abrupt decrease in the platelet count in response to viral infections is mediated by enhanced platelet destruction/clearance as a consequence of their activation [reviewed in (72)]. Data from several sources have identified a reduction in the platelet count in COVID-19 patients, where it is commonly associated with disease severity and increased risk of mortality (3, 5, 74–76). It has now been shown that SARS-CoV-2 and platelet interaction triggers programmed cell death, as defined by the presence of necroptosis and apoptosis markers in platelets from COVID-19 patients (77). Indeed, markers of platelet apoptosis, such as mitochondrial inner membrane depolarization, cytosolic calcium concentration, and PS exposure, were increased in COVID-19 patients in the ICU compared with non-ICU COVID-19 patients and healthy controls, demonstrating an association between severe COVID-19 and platelet apoptosis (78). In critically ill COVID-19 patients, these markers were also positively correlated with D-dimer levels and incidence of thrombotic complications (78). These clinical findings were corroborated by *in vitro* studies where isolated platelets from healthy donors stimulated with sera from severe COVID-19 patients resulted in platelet apoptosis mediated by immunoglobulin G (IgG) fractions from COVID-19 (78). These findings may explain the thrombocytopenia and the thrombotic phenotype observed in COVID-19 patients.

Platelets have been shown to sense and respond to viral components as part of the immune response [reviewed in (79)]. The literature review points out that platelets express a wide range of receptors on their surface which may mediate the binding and uptake of viral pathogens during influenza, HIV, and dengue infection (79–81). For example, during influenza infection, viral particles were detected inside platelets where they colocalized with Toll-like receptor 7 (TLR7) in the lysosomes (80). In the same way, it has been demonstrated that single-stranded RNA viruses activate the TLR7 receptor in human platelets leading to α-granule release and translocation of P-selectin to the cell surface (80, 82). Interestingly, blood samples or isolated platelets from COVID-19 patients examined either by different microscopy techniques or RNA-sequencing analysis revealed that SARS-CoV-2 particles are either attached to or inside platelets (76, 77). However, whether the SARS-CoV-2 virus can enter platelets *via* binding ACE2 and TMPRSS2 receptors is still debated since there is no consensus if human platelets express both receptors (76, 83, 84). The fact remains that SARS-CoV-2 interacts and possibly enters platelet, in spite of the concern whether ACE2 plays a role in this interaction. Either binding of SARS-CoV-2 to platelets or the SARS-CoV-2 RNA uptake has been shown to enhance platelet activation (76, 83, 84). Uptake of SARS-CoV-2 virions leads to morphological changes in platelets with microparticles shedding and content release (77). In fact, increased levels of platelet-derived extracellular vesicles (EVs) have been previously described in the plasma of COVID-19 patients (84). Indeed, other significant laboratory findings, including increased mean platelet volume (MPV) and increased αIIbβ3 activation and P-selectin expression, corroborate that platelets from COVID-19 patients show a hyperactive phenotype (76, 85, 86). Once activated, platelets secrete a vast repertoire of bioactive molecules from their intracellular granules that play essential roles not only in thrombus formation, such as ADP and thromboxane A_2_ (TXA_2_), but also in inflammation [e.g., pro-inflammatory cytokines; reviewed in (87)]. The activation process also results in a dramatic reorganization of the platelet membrane, involving the exposure of new receptors, including glycoprotein αIIbβ3, which enables fibrinogen binding and allows platelet aggregation, and the translocation of P-selectin to the platelet surface (88, 89).

Similar to what has been observed for other viral infections, including influenza, HIV, and dengue (90–93), there is an increased P-selectin expression during clinical infection with SARS-CoV-2 (76, 83, 94). P-selectin surface expression is increased in COVID-19 patients compared to healthy controls. Whether or not this finding could be related to the disease severity has remained elusive so far (76, 83, 94). P-selectin expression was also found to be increased in platelets from COVID-19 patients upon stimulation (83). Interestingly, Spike protein was found to induce integrin activation and P-selectin expression in platelets isolated from healthy donors even in the absence of platelet agonists (76). In addition to P-selectin, increased CD63 expression was also observed in platelets from SARS-CoV-2 infected patients (83, 94). Both P-selectin and CD63 are increased on the surface of platelets after granule release and are therefore considered markers of platelet activation (95). In line with the observation that CD63 modulates platelet spreading on immobilized fibrinogen (96), it has been demonstrated that platelets from COVID-19 patients exhibit greater adhesion and spreading on fibrinogen and collagen (83). Additionally, *in vitro* studies where platelets from healthy donors were incubated with SARS-CoV-2 or Spike protein showed an enhanced spreading on immobilized fibrinogen and clot retraction (76).

P-selectin is also considered an essential receptor for forming platelet leukocyte aggregates since the binding to its counter-receptor (P-selectin glycoprotein ligand 1; PSGL-1) on the leukocyte surface enables the adhesion of activated platelets to leukocytes (97–99). Platelet-neutrophil and platelet-monocyte aggregates, which are considered a more sensitive indicator of *in vivo* platelet activation, were significantly elevated in COVID-19 patients compared to healthy individuals (83, 85, 94, 100, 101). A similar outcome was observed when the whole blood of healthy controls treated with SARS-CoV-2 and Spike protein *in vitro* resulted in an increased proportion of platelet-leukocyte aggregates (76). It should be mentioned that increased levels of circulating platelet-leukocyte aggregates have been considered a marker of a prothrombotic state and are associated with several thrombotic diseases (102).

Another reason to believe that SARS-CoV-2 infection induces greater platelet reactivity is that platelets from COVID-19 patients are more sensitive to aggregation in response to low concentrations of agonists, such as thrombin and collagen, compared to healthy individuals. This hyperreactivity seems to be more pronounced in severe patients than in non-severe patients (76, 83). There is evidence that increased platelet response during SARS-CoV-2 infection is, at least in part, mediated by increased MAPK signaling pathway since the phosphorylation of ERK1/2, p38, and eIF4E, was found to be upregulated in platelets from COVID-19 patients (76, 83). Phosphorylated MAPK was detected in platelets from healthy donors after stimulation with SARS-CoV-2 or Spike protein (76). It was previously demonstrated that activation of MAPK in platelets induces the activation of a cytosolic phospholipase A2 (cPLA2), which results in thromboxane synthesis (103). Interestingly, phosphorylation of cPLA2 was increased in COVID-19 patients both at baseline and following platelet activation *in vitro* (83). It is well-known that increases in cPLA2 phosphorylation activate cPLA2 activity, upregulating thromboxane production (103). Indeed, it was found that plasma levels of TXA_2_ and thromboxane B_2_, a metabolite from platelet TXA_2_ synthesis, are higher in severe COVID-19 patients (83, 94). Although TXA_2_ can be secreted by cell types other than platelets, such as endothelial cells (ECs) and macrophages, it was observed that platelets from severe COVID-19, but not mild/asymptomatic subjects, had increased TXA_2_ synthesis (94).

The platelet hyperactivation observed in COVID-19 patients may be triggered by damaged endothelium. It is well-known that exposure of platelets to components of the subendothelial matrix might lead to platelet activation and aggregation in an attempt to repair the injured tissue (104, 105). Under normal circumstances, platelets circulate in a quiescent, non-adhesive state; however, they can be rapidly activated by subendothelial matrix components and soluble agonists such as ADP, TXA_2_, and thrombin (104). Another important step of platelet activation is the loss of lipid asymmetry, favoring the exposure of anionic lipids such as PS to the outer membrane. This rearrangement in the membrane phospholipids creates a procoagulant surface on platelets where the clotting factors can anchor and be activated, bursting thrombin formation (89). The thrombin generated cleaves fibrinogen into fibrin, which then interacts with activated platelets in order to stabilize the aggregates (89, 106). Notably, increased factor XII activity was detected in platelets isolated from COVID-19 patients, which was accompanied by a shortening in the activated partial thromboplastin time (APTT) measured in platelet-rich plasma (85). In other words, in the study conducted by Taus et al., platelets from COVID-19 patients show a procoagulant phenotype.

One of the most prominent characteristics of severe COVID-19 is the massive release of a wide range of cytokines, such as TNF, interferon γ (IFN-γ) and IL-1, IL-6, and IL-18, which characterizes the cytokine storm observed in SARS-Cov-2 infected patients (56–59, 107, 108). It is well-established that some of these inflammatory cytokines can have direct effects on platelet function, contributing to their thrombotic propensity (109–111). In line with the observation that IL-6 and IL-1β affect platelet function, it has been demonstrated that both cytokines can enhance agonist-induced platelet aggregation (109, 110). Additionally, there is robust evidence that IL-6 and IL-1β foster platelets hyperactivation and spreading (111). Interestingly, plasma from COVID-19 patients triggered platelet activation *in vitro*, as demonstrated by a significant increase in platelet P-selectin and CD63 surface translocation and platelet-leukocyte aggregates formation (94, 112), which was dampened by pretreatment with IL-6 receptor inhibitor (113). The resultant platelet hyperactivation may be evoked by inflammatory mediators present in the plasma of COVID-19 patients.

## Monocytes/Macrophages

Severe SARS-CoV-2 infection is characterized by an excessive inflammatory response with the release of a large number of pro-inflammatory cytokines (57–59, 107). Similar findings have been documented in patients infected with the severe acute respiratory syndrome (SARS) and the Middle East respiratory syndrome (MERS) coronaviruses, where hypercytokinemia was considered the main cause of morbidity (114). There is emerging evidence that infiltration of pro-inflammatory monocytes is the critical mediator of the hyperinflammatory response following SARS-CoV-2 infection, being responsible for the cytokine storm observed during the acute phase in severe cases (115–117). Single-cell transcriptomic analysis of bronchoalveolar fluid from COVID-19 patients revealed an increased number of mononuclear phagocytes (MNPs) in severe patients compared to those with mild symptoms or healthy controls (115). The MNP composition in severe patients showed a lower proportion of tissue-resident alveolar macrophages and a higher proportion of inflammatory monocyte-derived macrophages (115). On the other hand, single-cell RNA sequencing of peripheral blood from COVID-19 patients revealed a reduced number of non-classical and intermediate monocytes and impaired immune response by myeloid cells with reduced expression of cytokines, such as IL-6, TNF, and IL-1β (118–120). Considering that cytokine plasma levels are enhanced in SARS-CoV-2 infected patients, those more recent findings suggest a tissue origin of the plasma cytokines. Moreover, circulating levels of pro-inflammatory cytokines were found to be significantly higher in severe COVID-19 patients than in those with mild symptoms, suggesting that this intense cytokine production is positively associated with disease severity (56, 57, 59, 107, 108).

Similar to what has been reported in other respiratory viral diseases, the infection of airway epithelial cells with SARS-CoV-2 triggers the innate immune response, resulting in the activation of monocytes, macrophages, and dendritic cells (121). Several lines of evidence have suggested that monocytes/macrophages are attracted to the alveolar space in response to the viral infection elicited by SARS-CoV-2, where they secrete a wide range of pro-inflammatory cytokines and chemokines, including IL-1β and IL-6 and TNF, contributing to the hyperinflammatory and hypercoagulable phenotypes observed in severe COVID-19 patients (122, 123). In critically ill COVID-19 patients, plasma levels of pro-inflammatory cytokines, in particular the aforementioned IL-1, IL-6, and TNF-α, were found to be upregulated (57–59, 107). Higher levels of IL-6 seem to be associated with the severity of the disease (56, 107, 108), even though it has been reported that circulating IL-6 levels are lower in COVID-19 patients compared to what is observed in other pathological conditions, such as acute respiratory disease syndrome (ARDS) and cytokine release syndrome (CRS) (124).

In fact, it is well-known that activation of coagulation and intravascular coagulation that occurs during sepsis are mainly mediated by the expression of TF on monocytes in response to a number of different inflammatory extracellular stimuli (125–127). In such conditions, monocytes and macrophages respond with increased expression and release of TF (125–127). Likewise, increased TF activity was detected in EVs isolated from plasma of COVID-19 patients compared with healthy controls (128). Since Rosell et al. could not determine the origin of the TF-positive EVs, they speculate that they are derived from activated monocytes and endothelial cells.

The exposition of TF to the blood allows it to bind plasma factor VIIa forming a complex that initiates blood coagulation. The sequential proteolytic cleavage of coagulation factors culminates in the generation of thrombin, which can promote fibrin clot formation and platelet activation and enhance the pro-inflammatory response (53). Although monocytes are considered the primary source of TF in inflammatory states, especially during bacterial sepsis (127), the role of monocytes in virus-induced hypercoagulability remains limited. *In vitro* studies have shown that cytomegalovirus and influenza virus can upregulate TF expression on infected human monocytes, giving these cells a procoagulant phenotype (129). *In vivo* studies have correspondingly demonstrated a correlation between TF expression on monocytes and coagulopathy in HIV infection (130). In COVID-19, EV-TF activity was positively correlated with D-dimer, prothrombin time, and von Willebrand factor levels, indicating a link between TF-positive EVs, coagulation, and endothelial activation, which may contribute to thrombosis in COVID-19 patients (128).

It was demonstrated that TF expression by monocytes in COVID-19 is mediated by the crosstalk between monocytes and platelets (94). The platelet-monocyte interaction is also observed in other viral infections, including influenza (131), and during SARS-CoV-2 infection, it is mediated by P-selectin (83, 94). Of note, there is evidence of a positive and significant correlation between platelet-monocyte aggregates and P-selectin expression on the platelet surface in COVID-19 patients, suggesting that activated platelets interact with monocytes and, thereby, induce the expression of TF (85, 94). Indeed, monocyte TF expression appears to be higher in critically ill COVID-19 patients compared to non-infected individuals (94). Interestingly, monocytes from healthy volunteers exhibited increased TF expression after incubation with platelets from COVID-19 patients (94). Not surprisingly, severe COVID-19 patients showed a higher level of platelet-monocyte aggregates than asymptomatic/mild infected subjects and healthy controls (94).

Research over the past decade has linked anti-viral responses and virus-associated illnesses to the NLRP3 inflammasome activation (132–134). It has been recently reported that the SARS-CoV-2 virus can elicit such response. However, to date, the exact mechanisms by which SARS-CoV-2 activates NLRP3 inflammasome remain unclear (135–137). NLRP3 inflammasome activation mediates the autocatalytic activation of caspase-1, leading to maturation and secretion of IL-1β and IL-18, and has recently been implicated in the pathogenesis of thrombosis (138–140). Previous research has observed that IL-1β elicits tissue factor expression in monocytes-macrophages (141) and enhances the production of plasminogen activation inhibitor (142), resulting in a hypercoagulable state. High concentrations of active caspase-1 were detected in the sera of COVID-19 patients, and this increase was found to be more prominent in patients with the severe form (137). Additionally, active intracellular caspase-1 was detected in PBMCs obtained from COVID-19 patients, and the maintenance of these cells in culture resulted in increased active caspase-1 and IL-1β in the supernatant (137). *In vitro* studies have shown that infection of human monocytes with SARS-CoV-2 leads to an increase in procaspase-1 cleavage and IL-1β production, which can be impaired by NLRP3 inhibitors such as MCC950 and glyburide (136, 137). Together, these findings strongly support the participation of NLRP3 inflammasome in the innate immune responses to SARS-CoV-2. Upon activation, the NLRP3 recruits and interacts with apoptosis-associated speck-like protein containing a caspase recruitment domain (ASC) which in turn interacts with procaspase-1 (138). During this event, ASC forms aggregates known as “specks” in the cytosol, which are considered a readout for inflammasome activation (143). Interestingly, it was recently reported that SARS-CoV-2 induces ASC speck formation in human monocytes, which can be inhibited by MCC950 (137). In addition, NLRP3 and ASC specks were visualized by fluorescence microscopy in PBMCs from COVID-19 patients, indicating active inflammasomes in cells from patients infected with SARS-CoV-2 (137). Indeed, NLRP3 and ASC specks were also observed in postmortem lung tissues from COVID-19 patients (137). Immunofluorescence analysis of the autopsy lung samples revealed higher numbers of NLRP3 and ASC speck in samples from COVID-19 patients compared to controls (137).

Another feature of inflammasome activation is the cleavage of Gasdermin D (GSDMD) mediated by caspases, which triggers a form of programmed cell death termed pyroptosis (138). Human monocytes infected with viable SARS-CoV-2 showed enhanced cleaved GSDMD, which suggests that SARS-CoV-2 induces pyroptotic cell death in human monocytes (136). Interestingly, it was demonstrated that following inflammasome activation, pyroptotic macrophages release TF that is required for coagulation activation (144).

There is evidence that cells undergoing a lytic cell death release their intracellular content, including lactate dehydrogenase (LDH). Flow cytometry analysis revealed that SARS-CoV-2 induced lytic cell death in human monocytes, which culminated in an increased concentration of LDH in the supernatant compared to non-infected cells (136). Both LDH and IL-1β are released as a result of inflammasome activation (59, 145, 146). A large number of clinical data collected from COVID-19 patients revealed high levels of circulating lactate dehydrogenase (LDH) and IL-1β in critically ill patients (56–58, 100, 147).

## Neutrophils

Neutrophil count was found to be higher in severe COVID-19 patients than in patients with less severe symptoms (2, 57, 75, 100, 148, 149). The increase in blood neutrophil levels is concomitant to the disease progression and severity (1), and it has been described as an indicator of poor outcome (56, 150). In particular diseases, including some types of cancer and myeloproliferative disorders, neutrophilia is considered a reliable marker of a prothrombotic state (151–153). Several prospective cohort studies have highlighted the association between the high number of circulating neutrophils and the occurrence of venous thromboembolism (VTE) in cancer patients (154, 155). Whereas, the number of neutrophils is increased, severe COVID-19 patients often experience a drop in the lymphocyte levels, which results in a high neutrophil to lymphocyte ratio (NLR) (100, 150, 156). High NLR, a recognized marker of systemic inflammation that has been considered a predictor of VTE in cancer patients, was also found to be an independent risk factor predicting COVID-19 severity (148, 157–160).

It has been experimentally demonstrated that, in addition to their increased number in the circulation, neutrophils from COVID-19 patients show decreased granularity, which suggests a pre-activated state, and increased ability to spontaneously form NETs (100, 101, 147, 149, 161). Previous studies have shown that upon activation or during NETosis, neutrophils release calprotectin (162, 163). Noteworthy, increased levels of circulating calprotectin have been observed in COVID-19 patients with either severe or moderate disease (164, 165). Serum and plasma levels of calprotectin were found to be positively correlated with disease severity and negatively correlated with oxygenation efficiency (164, 165). Recent evidence suggests that increased levels of calprotectin during COVID-19 are not only associated with a worse outcome but also with thrombogenic and pro-inflammatory phenotypes following SARS-CoV-2 infection. Indeed, it was demonstrated that calprotectin secreted by platelets from COVID-19 patients promotes endothelial activation, as defined by upregulation of coagulation-related and pro-inflammatory genes by ECs *in vitro* (166).

NETs are formed when neutrophils, upon activation, expel their nuclear content decorated with granule proteins [e.g., myeloperoxidase (MPO) and neutrophil elastase (NE)] to the extracellular milieu (167, 168). They were first recognized for their role in bacterial clearance, but there is recent evidence that NETs are part of the innate immune response during a viral infection, such as those caused by influenza and the respiratory syncytial viruses (167, 169, 170). Similar to the phenomena observed in pneumonia-associated ARDS, neutrophils from COVID-19 patients are more prone to form NETs (171–175). In fact, there is evidence that neutrophils infected with a virus respond to it by releasing NETs (170, 176, 177). Not surprisingly, neutrophils containing SARS-CoV-2 antigens were found in blood samples from a cohort of COVID-19 patients, where they were more efficient in forming NETs compared to healthy neutrophils (149). Additionally, it was demonstrated that viable SARS-CoV-2 could induce NET formation in neutrophils from healthy donors *in vitro* (149). In line with the observation that aPL antibodies stimulate neutrophils to release NETs (178) it was demonstrated that IgG isolated from COVID-19 patient serum enriched for aPL induces healthy neutrophils to form NETs to a similar extent compared to IgG samples obtained from patients with antiphospholipid syndrome (APS) (47).

An increasing number of studies have detected high circulating levels of NETs in COVID-19 patients (101, 147, 149, 161). As compared with samples from healthy individuals, the COVID-19 patient samples showed higher levels of cell-free DNA (100, 147), MPO-DNA complexes (100, 101, 147, 149, 161), and citrullinated histone H3 (100, 147), well-known markers of NET formation. These markers have been positively correlated with circulating D-dimer levels in SARS-CoV-2 infected patients sera, indicating an association between NETosis and a higher risk of thromboembolic events in COVID-19 (179). The excessive NET formation is also observed in several pathological conditions such as autoimmune and pulmonary diseases and thrombosis (180). It has been recognized by the literature that NETs exhibit a number of thrombogenic properties, including the ability to initiate the intrinsic pathway of coagulation, serving as a scaffold for the adherence of platelets and red blood cells, to degrade natural coagulation inhibitors, and finally, to exert antifibrinolytic effects (181). There is a growing body of literature that provides mechanistic insights into how NETs propagate inflammation and thrombosis. For example, DNA released from activated neutrophils, like other polyanionic molecules, elicits blood coagulation by amplifying factors XI and XII activation (182). In severe sepsis patients, the cell-free DNA levels in circulation correlate with increased thrombin generation (182). Histones, another main component of NETs, exert prothrombotic activity by enhancing thrombin generation either by activating platelets (183) or by impairing protein C activation (184). At the same time, the serine protease neutrophil elastase (NE) acts, together with extracellular nucleosomes, to inactivate the tissue factor pathway inhibitor (TFPI), thus resulting in increased procoagulant activity (185). Together, these studies support the hypothesis that the hypercoagulable state observed in COVID-19 patients is, at least partially, mediated by neutrophil activation and NET formation. This notion is further supported by the evidence that MPO-DNA levels were positively correlated with thrombin-antithrombin (TAT) levels in SARS-CoV-2 infected patients (161).

Neutrophils can also interact with activated platelets at sites of inflammation, thereby facilitating NET formation. The interactions between neutrophils and platelets are mainly mediated by the binding of platelet adhesion molecules or glycoproteins to their ligands on the neutrophil surface (99, 186). The formation of platelet-neutrophil aggregates not only contributes to NET-mediated virus clearance (187), but also propagates thrombus formation (188). Interestingly, neutrophils from COVID-19 patients were found to be decorated with platelets, especially in severe patients (100). Indeed, higher levels of circulating platelet-neutrophil aggregates were detected in COVID-19 patients compared with healthy adults (100, 101). The crosstalk between neutrophils and platelets relies not only on cell-to-cell communication but also on secreted substances. Upon activation, platelets secrete a number of molecules, such as high mobility group box 1 (HMGB1) and platelet factor 4 (PF4), capable of modulating the activation of neutrophils, thus triggering NET formation (189, 190). *In vitro* studies revealed that platelet-rich plasma from COVID-19 patients stimulates neutrophils from healthy donors to increase the expression of TF and to release NETs (161). These TF-bearing NETs also showed a procoagulant activity indicated by the high levels of TAT complex, a parameter that reflex a hypercoagulable state (161).

Another prominent characteristic of COVID-19 is the extensive neutrophil infiltration in the pulmonary capillaries. Different groups have been describing a robust neutrophil infiltration in the pulmonary capillaries in COVID-19 revealed by autopsy of lung samples from patients infected with SARS-CoV-2 (101, 156, 191–193). Other respiratory viruses, including SARS-CoV and MERS-CoV, are also associated with neutrophil infiltration at sites of infection and development of ARDS (194, 195). Indeed, the ARDS is characterized by increased neutrophil infiltration and accumulation in the alveoli in response to pro-inflammatory cytokines and chemokines produced by an exuberant immune response (196). The neutrophil-attracting chemokines CXCL2 and CXCL8 were shown to be overexpressed by human epithelial cells infected with SARS-CoV-2 *in vitro* (197). Immunofluorescence analysis of lung sections from COVID-19 patients revealed not only a robust neutrophil infiltration but also typical NETs structures (101, 149). Neutrophils within the lung microvasculature were found to be trapped, associated with platelets, in fibrin meshworks (101, 156) and these clots may contribute to the disease severity. In this context, microvascular thrombi containing NETs associated with platelets and fibrin have been found in the lung, kidney, and heart of COVID-19 patients, as demonstrated by post-mortem histopathological analysis (17).

## Therapeutic Perspectives

COVID-19 is generally a respiratory infection; however, some patients develop with non-respiratory symptoms. Research groups around the world have observed a hypercoagulability and an increased risk for venous thromboembolism and arterial thrombosis in SARS-CoV-2 infected patients (4–7, 15, 16, 57). There is, indeed, an association between thromboembolic events and higher mortality among COVID-19 positive patients (11–14). In this review, we summarized the main findings regarding the cellular mechanism of COVID-19-associated coagulopathy that may provide evidence for the use of conventional therapy for thrombosis. In fact, some studies have been conducted to validate the impact of prophylactic or treatment dose anticoagulation, as well as antiplatelet therapy on both thromboembolism incidence and mortality in COVID-19 patients; nevertheless, data are still controversial.

Data from the HEP-COVID study showed that therapeutic anticoagulation with low molecular weight heparin (LMWH) or unfractionated heparin (UFH) was associated with a reduction in venous thromboembolism (VTE), arterial thromboembolism (ATE) in a cohort of non-severe or non-ICU COVID-19 patients (198). In addition, therapeutic heparins doses significantly reduced the mortality from all cause in non-ICU patients, which was not observed in patients admitted to the ICU (198). However, data from the INSPIRATION trial showed no benefit of an intermediate dose of LMWH in preventing VTE or ATE compared with the prophylactic dose of LMWH in ICU patients (199). Similarly, another randomized clinical trial (RAPIDTrial) did not detect any significant reduction in the primary composite of death, mechanical ventilation, or ICU admission in patients receiving a therapeutic dose of LMWH or UFH compared with a prophylactic dose of heparins, although it was associated with a lower incidence of death at 28 days (200). Early initiation of anticoagulation with prophylactic doses of heparin or enoxaparin within 24 h of hospital admission was associated with a decreased risk of 30-day mortality compared with no anticoagulation (201).

A recent retrospective study found that therapeutic anticoagulation either in combination with antiplatelet therapy or alone was associated with improved outcomes and decreased mortality in hospitalized COVID-19 patients in comparison to patients receiving prophylactic anticoagulation. The concomitant prophylactic anticoagulation and antiplatelet therapy were associated with a significantly lower rate of invasive mechanical ventilation compared with prophylactic anticoagulation alone (202). In line with these observations, a single-center retrospective study demonstrated an association between prophylactic anticoagulation and decreased in-hospital mortality (203).

An observational cohort study using an online multicenter international registry [Health Outcome Predictive Evaluation Registry (HOPE-COVID-19)] for patients with laboratory-confirmed SARS-CoV-2 observed no difference in embolic events and the need for mechanical ventilation between patients receiving antiplatelet therapy and those without; however, the duration of mechanical ventilation for those using antiplatelet therapy was significantly shorter (204). Multivariable regression analysis revealed that antiplatelet therapy during hospitalization was associated with a lower risk of mortality, even among critically ill COVID-19 patients (204). Comparably, a recent retrospective, observational cohort study analyzed the data from COVID-19 patients who were taking daily aspirin prior to hospitalization and concluded that pre-admission aspirin therapy was associated with a better in-hospital outcome, in spite of no difference in mortality rate compared to patients who had not received aspirin (205). A multicenter retrospective study observed that aspirin, administered within 24 h or in the 7 days before hospitalization, did not decrease the rate of thrombosis; however, it was associated with a lower risk of ICU admission, mechanical ventilation, and in-hospital death (206).

Research over the past year has provided evidence that the innate immune system plays a critical role in patients' response to SARS-CoV-2 infection. It is becoming increasingly clear that some COVID-19 patients develop a hyperinflammatory syndrome resembling cytokine storm syndromes, which in turn drives the ARDS observed in these patients (56–59, 107, 108). The massive cytokine release in response to SARS-CoV-2 infection raises the possibility that pro-inflammatory cytokines may be a therapeutic target in COVID-19. Thus, immunomodulatory drugs have been proposed as potential therapies for the treatment of COVID-19 since they may mitigate the effects of the hyperinflammatory response. Indeed, several clinical trials are currently in progress to evaluate the benefits of using immunomodulators in COVID-19, and preliminary studies have been already published for anakinra, an IL-1 receptor antagonist that blocks the activity of IL-1α and IL-1β, canakinumab, a monoclonal antibody targeting IL-1β and tocilizumab, a monoclonal antibody that specifically targets IL-6 receptor (207–210). The findings of these studies are summarized in Table 1.

**Table 1:** Ongoing clinical trials for immunomodulators in COVID-19.

	**Ongoing clinical trials**	**Main findings in COVID-19**
**Targeting IL-1β**		
IL-1 receptor antagonist	NCT04330638 NCT04324021	5 mg/kg (i.v.) of anakinra twice a day; significant increase in survival at 21 days compared with SOC (90 vs. 56%) (208). 100 mg (s.c.) anakinra twice a day for 72 h, then 100 mg (s.c.) daily for 7 days; significant reduction in a composite outcome of mortality and/or intensive care unit admission compared with SOC (209). 300 mg (i.v.) once a day for 5 days, then 200 mg once a day for 2 days, and then 100 mg for 1 day; significant clinical improvement (no deaths, significant decreases in oxygen requirements and more days without invasive mechanical ventilation) compared with SOC (207). 100 mg (s.c.) anakinra once a day for 7–10 days; significant changes in laboratory values (increased lymphocyte count; decreased IL-6 and CRP plasma levels) on the first 7 days of treatment; significant reduction in WHO-CPS and SOFA scores; lower incidence of respiratory failure; significant reduction in 28-days mortality compared with SOC (211).
IL-1β neutralizing Ab	NCT04362813 NCT04510493	150 mg (s.c.) of canakinumab on days 1 and 7; significant clinical improvement in ventilation regimes (significant increase in PaO_2_:FiO_2_ and reduction in lung damage); significant decreases in immune/inflammation markers; significant increase in survival at 60 days compared with SOC (90.0 vs. 73.3%) (210).
**Targeting IL-6**		
IL-6 receptor antagonist	NCT04330638 NCT04435717 NCT04377750	Tocilizumab was administered in the first 2 days in ICU; lower risk of in-hospital death compared with SOC (HR, 0.71; 95% CI, 0.56–0.92) (212). 8 mg/kg (i.v.) of tocilizumab on day 1 and 400 mg (i.v.) on day 3 (conditional on patients' oxygen requirement); lower risk of NIV, MV, or death by day 14; faster decreases in immune/inflammation markers compared with SOC (213). 8 mg/kg (i.v.) of tocilizumab, single dose (not exceeding 800 mg); no difference in disease worsening at day 14 compared with SOC (18 vs. 14.9%); no difference in MV or death compared with SOC (214).
IL-6 neutralizing Ab	NCT04330638 NCT04348500 NCT04343989	No results reported until September 30, 2021.
**Targeting inflammasome**		
Selective NLRP3 inhibitor	NCT04382053 NCT04540120	No results reported until September 30, 2021.
**Targeting NETs**		
DNase	NCT04445285 NCT04359654 NCT04432987 NCT04402944	2.5 mg of dornase alfa nebulized twice a day; treatment regime varying from 3 to 25 days; decrease in FiO_2_ requirements (215). 2.5 mg of dornase alfa nebulized once a day for 3 days; clinical improvement (reduction in lung damage, increase in SpO_2_ and disappearance of dyspnea and coughing) (216).
Defibrotide	NCT04652115 NCT04348383 NCT04335201 NCT04530604	No results reported until September 30, 2021.

*SOC, Standard of care; WHO-CPS, WHO Clinical Progression Scale; SOFA, Sequential organ failure assessment; PaO_2_:FiO_2_, Pressure of arterial oxygen to fractional inspired oxygen concentration; HR, Hazard Ratio; CI, Confidence interval; NIV, Non-invasive ventilation; MV, Mechanical ventilation; FiO_2_, Fraction of inspired oxygen; SpO_2_, Oxygen saturation*.

Given that pro-inflammatory cytokines, inflammasome and NETs may contribute to the thromboembolic events observed in critically ill COVID-19 patients. It is, therefore, reasonable to speculate that these aforementioned immunomodulators can also ameliorate the COVID-19–related hypercoagulable state. Actually, immunomodulators have been used not only to treat autoimmune and autoinflammatory diseases but also to improve cardiovascular outcomes in patients with cardiovascular diseases. In the CANTOS trial (Canakinumab Anti-Inflammatory Thrombosis Outcomes Study), the treatment of patients with previous myocardial infarct with the monoclonal IL-1β-neutralizing antibody canakinumab reduced the risk of recurrent cardiovascular events (217). The IL-1β blockade has also been proven to be effective in reducing thrombosis under hypoxic conditions (139). In their experimental model, mice treated with specific antibodies against active IL-1β showed a reduction in coagulation markers, including D-dimer and prothrombin fragment 1+2, and developed smaller venous thrombi than IgG treated mice (139). Neutralizing IL-1 resulted in a prominent decrease in venous thrombogenesis in CD39-deficient mice (140). The deficiency of CD39 in mice results in a higher incidence of thrombosis, and the treatment with either a neutralizing IL-1β antibody or the IL-1 receptor antagonist anakinra resulted in reduced occurrence or size of thrombus in inferior vena cava (IVC) stenosis mice (140). In a 4T1 murine breast cancer model, the treatment with anakinra decreased not only the tumor growth but also the thrombosis occurrence in tumor-bearing mice (218). Given that IL-1β can be secreted as a result of NLRP3 inflammasome activation (138), it is rational to think that NLRP3 inhibitors can dampen the pro-inflammatory and prothrombotic effects elicited by IL-1β. In fact, genetic ablation of NLRP3 was able to curtail venous thrombosis in animals exposed to hypoxia (139). *In vitro* studies revealed that pretreatment of human platelets with a direct NLRP3 inhibitor significantly reduces platelet aggregation in response to low concentrations of collagen and ADP and impairs clot retraction (219). Of note, a recent phase 3 clinical trial showed that early initiation of anakinra treatment in hospitalized patients with moderate or severe COVID-19 resulted in a significant increase in the lymphocyte count concurrent to a decrease in circulating IL-6 and CRP levels on the first 7 days of treatment. In this study, the 10-day treatment with anakinra was associated with a lower incidence of respiratory failure and a significant reduction in 28-days mortality (211). Further assessment of thrombosis-related parameters in this or similar trials may help to support a role for IL1/ILR in COVID-19-associated hyperthrombotic state.

The humanized monoclonal anti-IL-6 receptor antibody tocilizumab (TCZ) is used to treat rheumatoid arthritis (RA) and recent clinical trials have been proposed its use, in combination with standard of care, in the treatment of COVID-19 patients. Apart from the anti-inflammatory activity in RA, some trials also showed significant benefits with TCZ in terms of reducing hemostatic parameters associated with thrombosis (220, 221). Patients with moderately to severely active RA treated with TCZ intravenous reported fast and sustained reductions in fibrinogen and D-dimer (220). Additionally, another cohort of RA patients receiving subcutaneous TCZ treatment experienced an improvement in endothelial function and decreased NETs formation (221).

It is known that released NET components contribute to thrombus formation as a result of their function on blood coagulation, platelet activation and/or endothelial activation (182–185, 222). One of the major components of NETs are histones, and they are known to promote endothelial cell activation (222). It has recently been shown that endothelial cell activation induced by histones can be inhibited by defibrotide, a mixture of oligonucleotides currently used in the treatment of hepatic veno-occlusive disease (223, 224). Interestingly, a recent study showed that endothelial cell activation induced by COVID-19 patients' serum could be partially inhibited by defibrotide *in vitro* (224). NETs, which play an important role in thrombus propagation and stabilization, can be degraded by nucleases, such as DNase 1 (225). Studies in experimental models of thrombosis have shown that intravenous administration of DNase 1 protected mice from DVT after IVC flow restriction (226). Markedly, DNase 1-treated mice were less susceptible to DVT than vehicle-treated mice (226). Similarly, DNase 1 has also been shown to protect mice from ischemia-reperfusion injury (227). A combination of intravenous and intraperitoneal injections of DNase 1 in mice subjected to transient middle cerebral artery occlusion (tMCAO) reduced the infarct size by about 40% and significantly improved stroke outcome (227). Tumor-bearing mice also benefit from DNase 1 treatment. Mice bearing 4T1 breast tumors and treated with DNase 1 prior to thrombosis induction were protected from the increased thrombus formation characteristic of this experimental model (228). However, it has been demonstrated that long-term systemic treatment with DNase 1 may be deleterious in mice models of sepsis and cancer-associated thrombosis (229, 230).

DNase is currently used in the treatment of cystic fibrosis, where it is administered to these patients by nebulization (231). Clinical trials currently in progress also propose the use of nebulized DNase 1 for the treatment of respiratory failure in COVID-19. Although it has been disclosed that aerosolized DNase 1 promotes a reduction in systemic inflammatory markers (232), it is not yet clear whether nebulized DNase 1 will have any effect on circulating levels of NET or NET-mediated prothrombotic state in COVID-19.

In addition to the drugs mentioned in this section, current approaches to COVID-19 therapeutics also include other compounds with the potential to ameliorate either inflammation or thrombosis triggered by SARS-CoV-2, such as colchicine, Bruton tyrosine kinase inhibitors and activated protein C. However, while some studies are in a more advanced stage of analysis, others are still in the theoretical field and thus are not discussed in detail (191, 233, 234).

## Concluding Remarks

**Figure 1:**
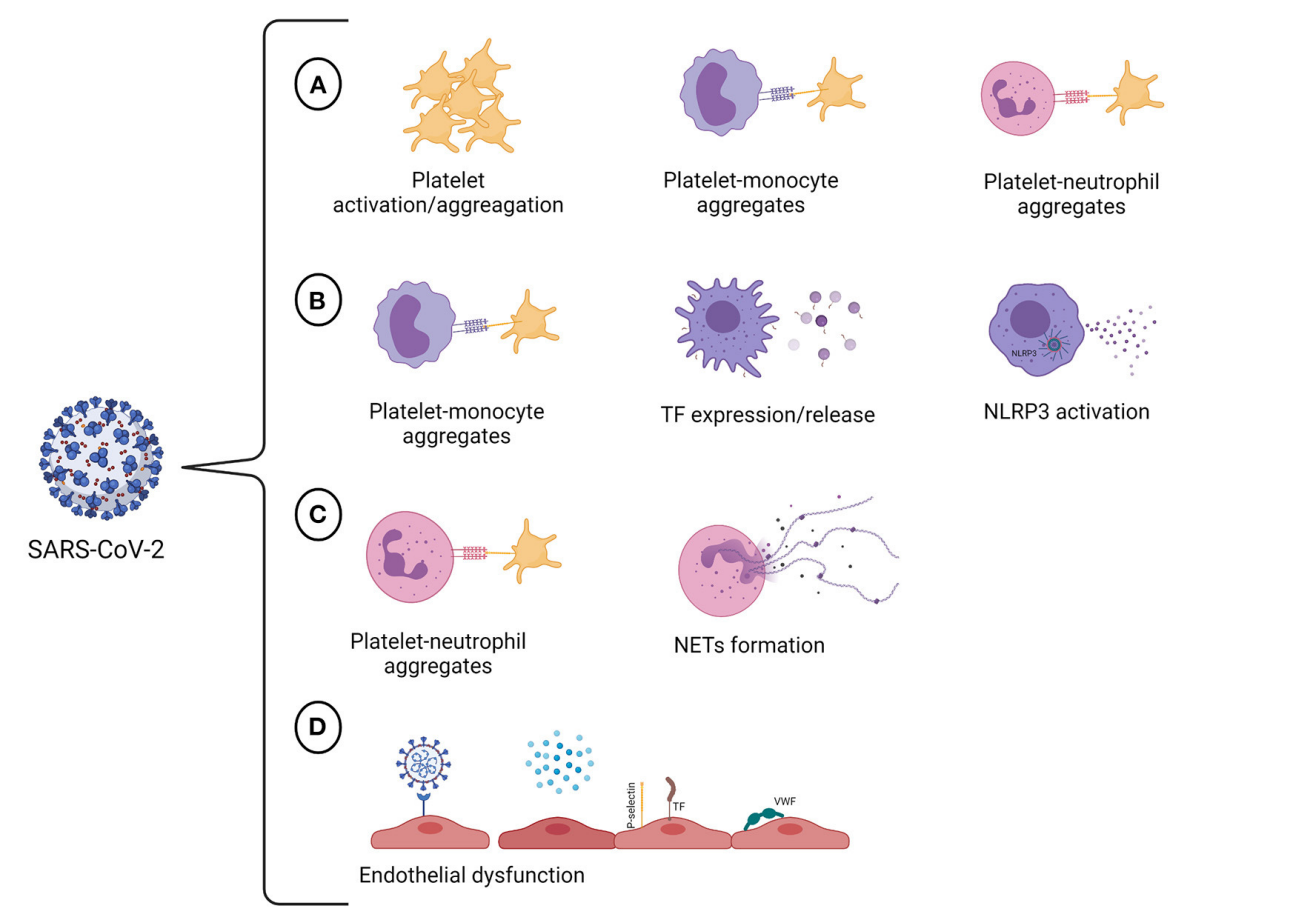
SARS-CoV-2 targets multiple vascular cells. **(A)** SARS-CoV-2 infection promotes hyperactivation of platelets. The consequences of platelet activation include reorganization of the platelet membrane, which enables fibrinogen binding and allows platelet aggregation, and the translocation of P-selectin to the platelet surface, enabling the formation of platelet-monocyte and platelet-neutrophil aggregates. **(B)** The crosstalk between platelets and monocytes can upregulate TF expression and release by monocytes, giving these cells a procoagulant phenotype. The infection of human monocytes with SARS-CoV-2 may activate the NLRP3 inflammasome, leading to an increase in procaspase-1 cleavage and IL-1β production and, therefore, resulting in a hypercoagulable state. **(C)** The interactions between neutrophils and platelets at sites of inflammation facilitate NET formation. It has also been proved that SARS-CoV-2 can induce NET formation in healthy neutrophils *in vitro*. NETs can propagate thrombus formation due to their thrombogenic properties, including the ability to initiate the intrinsic pathway of coagulation, to degrade natural coagulation inhibitors, and exert antifibrinolytic effects. **(D)** COVID-19 may trigger endothelial cell dysfunction either due to SARS-CoV-2 infection of endothelial cells or by pro-inflammatory cytokines that are produced in response to viral infection. The activation of endothelial cells results in the loss of the antithrombotic phenotype of the endothelium, occasioning the activation of the coagulation cascade, platelets and complement system in the vasculature. Created with BioRender.com.

Severe COVID-19 elicits an inflammatory-related hyperthrombotic state that significantly contributes to the fatal outcome. COVID-19-associated thrombosis is a complex process that seems to engage different vascular cells, including endothelial cells, platelets, monocytes, and neutrophils, that contribute to the establishment of the prothrombotic state (Figure 1). Remarkably, heterotypic cell-cell associations seem to play a pivotal role in amplifying the inflammatory/prothrombotic response not only during SARS-CoV-2 infection but also after recovering. Recent studies have shown a persistent prothrombotic phenotype in convalescent COVID-19 patients (235–237). It has been discussed that endothelial cell activation triggered by SARS-CoV-2 is sustained up to 10 weeks following acute infection (236). This may be responsible for the procoagulant state observed in convalescent COVID-19 patients (236, 237). Indeed, a higher level of D-dimer was detected in patients recovering from COVID-19, while other coagulation and inflammation markers were at basal levels (237). Besides the aforementioned factors, the assessment of NET markers may also be beneficial in the management of long-term post-COVID-19 patients since it was demonstrated that circulating NET remnants are increased in individuals up to 2 years after the diagnosis of venous thromboembolism (238). Although less is known about the long-term consequences, there is evidence that endothelial activation, low-grade inflammation, and hypercoagulability may persist in post-acute COVID-19. Thus, it is crucial to establish strategies to identify and monitor patients with a high risk of developing post-acute COVID-19 syndrome.

**Figure 2:**
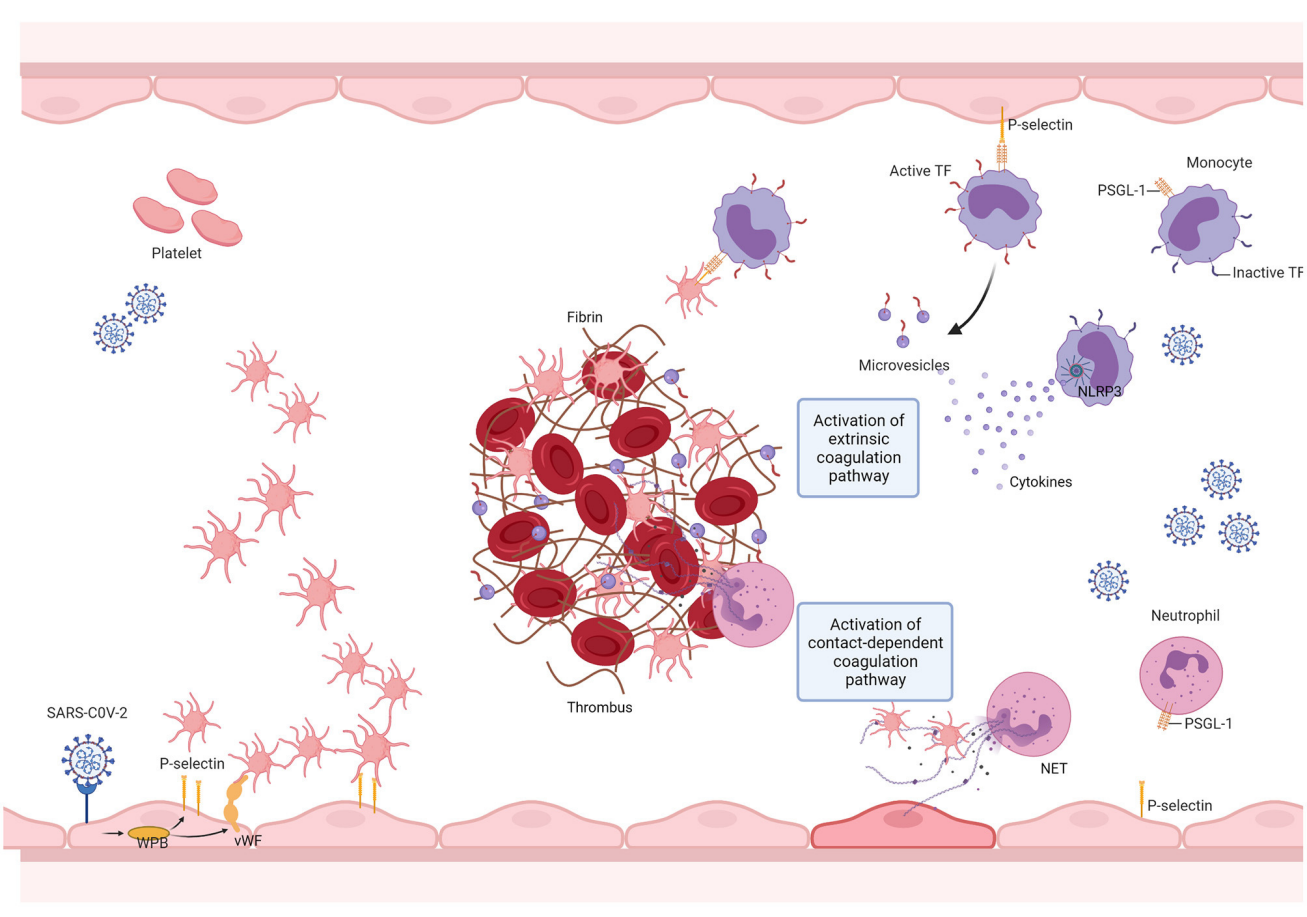
SARS-CoV-2 promotes thrombus formation by engaging multiple vascular cells. The SARS-CoV-2 infection triggers the innate immune response, resulting in the activation of monocytes, which in turn elicit a pro-inflammatory cytokine storm. This results in neutrophil recruitment, endothelial cell, and platelet activation. Endothelial cell activation is followed by the release of von Willebrand factor (VWF) and adhesion molecules, leading to platelet recruitment and activation. Platelet activation results in the exposure of new receptors, including glycoprotein αIIbβ3, which enables fibrinogen binding and allows platelet aggregation, and the translocation of P-selectin to the platelet surface, enabling the formation of platelet-monocyte and platelet-neutrophil aggregates. The interaction between platelets and monocytes and pro-inflammatory cytokines upregulate tissue factor (TF) expression and release it into microvesicles. TF then binds to coagulation factor VII (FVII), activating coagulation. SARS-CoV-2 infection may activate the NLRP3 inflammasome, leading to an increase in IL-1β production and, therefore, resulting in a hypercoagulable state. SARS-CoV-2 mediated cytokine storm promotes sustained neutrophil recruitment and activation, culminating in neutrophil extracellular trap (NET) formation, which fosters thrombus formation. Created with BioRender.com.

Taken together, we conclude that, as seen with other pathological states such as cancer, the prothrombotic condition observed in COVID-19 is a multifactorial and complex process (Figure 2). Further evaluation of additional mechanisms may help delineate novel pharmacological strategies and determine how COVID-19-associated thrombosis can contribute to the multiorgan dysfunction that characterizes this disease (239).

## Author Contributions

DM: writing—original draft preparation and editing. DM and RM: figure design. EH, PB, and RM: writing—review and editing and funding acquisition. All authors read the final draft and approved the manuscript for submission.

## Funding

The Brazilian National Council for Scientific and Technological Development (CNPq) under Grants 309946/2018-2 and 311686/2019-2, The State of Rio de Janeiro Research Foundation (FAPERJ) under Grants E-26/202.871 and E26.200.992/2021, The State of Minas Gerais Research Foundation (FAPEMIG) Grant APQ-02720-21, and the Coordination for the Improvement of Higher Education Personnel (CAPES) under Grants 88887.506989/2020-00 and 23038.009431/2021-42 supported this work.

## Conflict of Interest

The authors declare that the research was conducted in the absence of any commercial or financial relationships that could be construed as a potential conflict of interest.

## Publisher's Note

All claims expressed in this article are solely those of the authors and do not necessarily represent those of their affiliated organizations, or those of the publisher, the editors and the reviewers. Any product that may be evaluated in this article, or claim that may be made by its manufacturer, is not guaranteed or endorsed by the publisher.
